# Accurate Medical Vial Identification Through Mixed Reality: A HoloLens 2 Implementation

**DOI:** 10.3390/electronics13224420

**Published:** 2024-11-11

**Authors:** Bahar Uddin Mahmud, Guan Yue Hong, Afsana Sharmin, Zachary D. Asher, John D. Hoyle

**Affiliations:** 1 Department of Computer Science, Western Michigan University, Kalamazoo, MI 49008, USA; 2 Department of Mechanical and Aerospace Engineering, Western Michigan University, Kalamazoo, MI 49008, USA; 3 MD School of Medicine, Western Michigan University Homer Stryker, Kalamazoo, MI 49008, USA

**Keywords:** vial detection, barcode reading, mixed reality, medical vials, healthcare technology, patient safety, Microsoft HoloLens 2

## Abstract

The accurate identification of medicine vials is crucial for emergency medical services, especially for vials that resemble one another but have different labels, volumes, and concentrations. This study introduces a method to detect vials in real-time using mixed reality technology through Microsoft HoloLens 2. The system is also equipped with an SQL server to manage barcode and vial information. We conducted a comparative analysis of the barcode detection capabilities of the HoloLens 2 camera and an external scanner. The HoloLens 2 effectively identified larger barcodes when they were 20–25 cm away in normal lighting conditions. However, it faced difficulties in detecting smaller barcodes that were consistently detected by the external scanner. The frame rate investigation revealed performance fluctuations: an average of 10.54 frames per second (fps) under standard lighting conditions, decreasing to 10.10 fps in low light and further reducing to 10.05 fps when faced with high barcode density. Resolution tests demonstrated that a screen resolution of 1920 × 1080 yielded the best level of accuracy, with a precision rate of 98%. On the other hand, a resolution of 1280 × 720 achieved a good balance between accuracy 93% and speed. The HoloLens 2 demonstrates satisfactory performance under ideal circumstances; however, enhancements in detecting algorithms and camera resolution are required to accommodate diverse surroundings. This approach seeks to help paramedics make quick and accurate decisions during critical situations and tackle common obstacles such as reliance on networks and human mistakes. Our new approach of a hybrid method that integrates an external Bluetooth scanner with the MR device gives optimal results compared to the scanner-only approach.

## 1. Introduction

The National Coordinating Council for Medication Error Reporting and Prevention defines medication error as any preventable event that may result in improper medication use or harm while the medication is under the control of healthcare professionals, patients, or consumers. These errors encompass all stages of medication management, from prescribing to administration. Medical vials include vital information on medications, dosage, and expiration dates. The incorrect identification of a vial can have serious consequences, such as incorrect administration of the medication and possible harm to patients [1]. On the other hand, some vials may look similar, which makes it difficult for medical professionals to distinguish them during emergencies. However, medicine vials should be prescribed to patients based on their illness and other relevant factors such as age and weight. Traditional barcode scanning systems usually rely on stable network connections, which may not always be available, especially in remote or crowded environments. When internet access is unavailable, correctly reading the barcode printed on a medicine vial with mixed reality technology offers a robust solution to reduce the risk of a wrong medicine being administered to a patient. Medication errors, particularly in high-pressure situations such as emergency medical services, can lead to severe consequences. A recent analysis of prehospital pediatric medication dosing errors highlighted the persistence of errors even with state-wide dosing aids like the MI-MEDIC card, with a significant portion of these errors occurring in medications requiring complex calculations (e.g., dextrose, glucagon, intranasal fentanyl, midazolam, and epinephrine) [2]. Such findings emphasize the need for more robust systems that ensure correct dosage calculations and aid in accurately identifying vials in real-time, especially when vials look alike. Our study introduces a mixed reality system using the Microsoft HoloLens 2 to address these challenges by detecting and identifying vials efficiently, reducing human error, and improving patient safety. Medication errors, especially in pediatric care, are a significant concern due to their high prevalence and potential severity. Research has shown that pediatric patients are particularly vulnerable to such errors, which often result from incorrect dosages and adult-formulated drugs in pediatric settings. These errors are notably frequent in emergency and intensive care environments, where the fast-paced nature of treatment can lead to mistakes in medication administration. A comprehensive review of medication errors in pediatrics emphasizes the need for enhanced preventive strategies, including electronic prescribing systems, barcoding, standardized dosage protocols, and improved reporting mechanisms to reduce the likelihood of such errors and improve patient outcomes [3]. This highlights the critical role of technology in addressing medication safety, making innovations like mixed reality systems for vial identification crucial for minimizing risks in healthcare settings. Medical vial identification is a crucial process in healthcare settings. Given the variability in vial sizes, labeling inconsistencies, and the fast-paced nature of medical environments, there is a high risk of human error in identifying vials. This is particularly critical, as mistakes in vial identification can lead to serious health complications. Previous studies have focused on barcode scanning technologies and image processing methods, but they often lack the necessary speed and precision required in medical contexts.

### 1.1. Scope of the Work

#### 1.1.1. Code Detection

According to Microsoft mixed reality documentation [4], to obtain the most effective QR code, for our case, barcode detection, it is necessary to adhere to several recommended guidelines. Codes require a quiet zone, which is a four-module-wide space around all sides of the code. This quiet zone should be free of written material. This margin guarantees that the code is interpreted accurately.

#### 1.1.2. Lighting and Backdrop

The quality of detection is greatly influenced by the lighting and backdrop, so it is good practice to ensure adequate contrast between the black and white modules under typical lighting conditions. When dealing with intense lighting conditions, such as very bright or very black backgrounds, enhancing detection rates can be achieved by decreasing and fine-tuning the contrast. This can be performed by reducing the white background color to a RGB value below 255.

#### 1.1.3. Size of the Code

**Figure 1.**
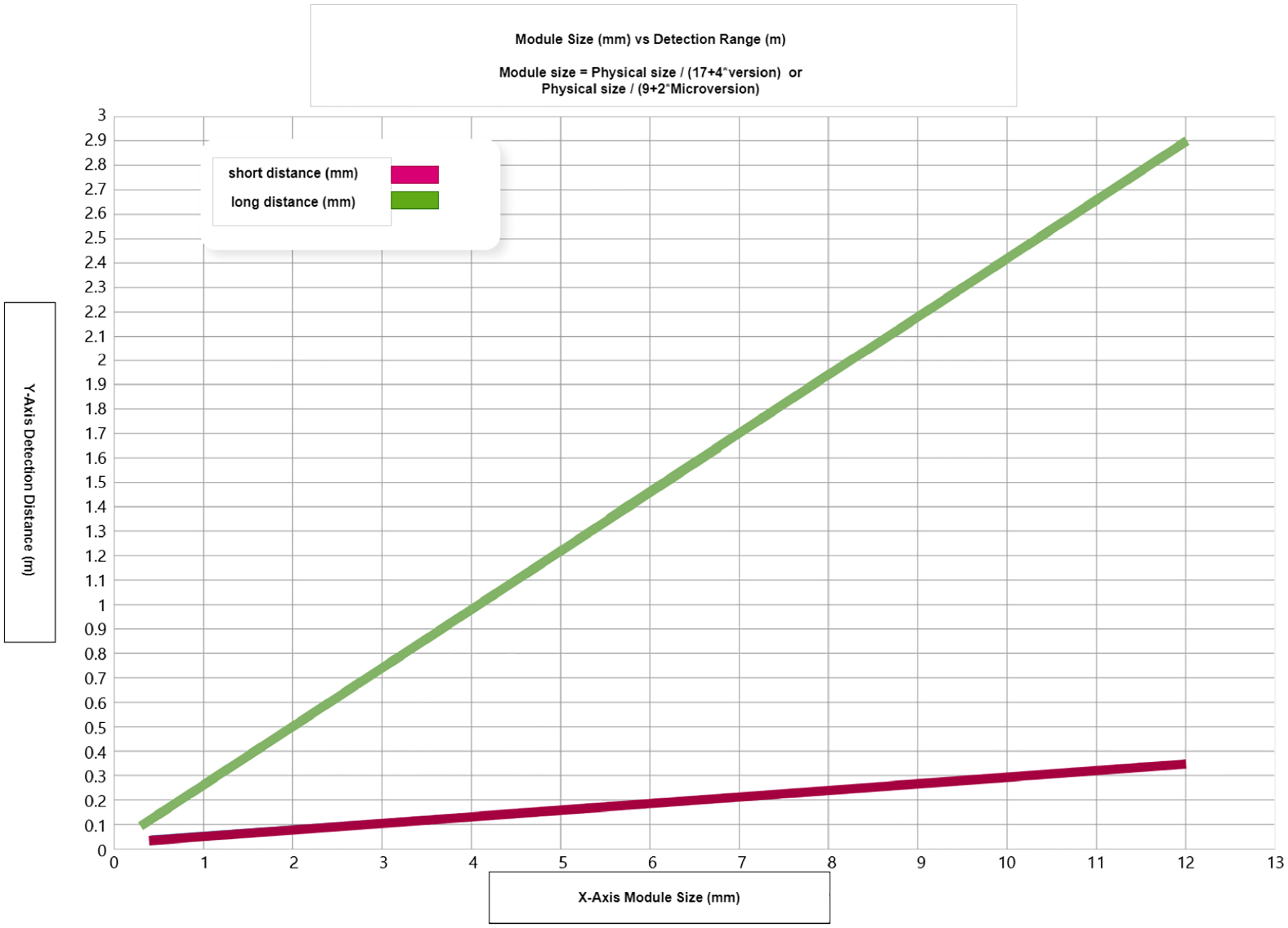
The trade-off between the distance and size of the code for optimal detection.

The dimensions of codes are also crucial. Windows mixed reality and HoloLens devices are incompatible with codes that have dimensions smaller than 5 cm on each side. When dealing with codes ranging from 5 to 10 cm, the device must be positioned at a shorter distance, and the detection process may require more time. The time required to detect a code is generally influenced by both its magnitude and proximity to the code. Approaching closer can alleviate problems related to size. As shown in Figure 1, the graph [5] illustrates the relationship between module size and detection distance for different versions of codes (for versions 1–10) and micro codes (for versions M1–M4). The formulas used in the figure calculate the module size by taking into account the version or microversion, which directly impacts the detection range.

#### 1.1.4. Distance and Angular Position

Both the distance at which an object can be detected and its precise angular position are crucial factors to consider. Proximity is crucial for small codes that measure less than 10 cm on the side. The minimum detection distance for version 1 QR codes, which have sizes ranging from 10 to 25 cm, is between 0.15 m and 0.5 m. The distance between the code and the detection device increases linearly as the size of the code increases. However, this distance is also influenced by the version of the code and the size of the module. In higher versions of the qr/barcode code, the modules are smaller, necessitating closer detection. Micro-qr codes can increase the range at which they can be detected. The best detection occurs within a range of ±45 degrees to guarantee sufficient resolution. Additional factors to consider are refraining from using barcodes on curved surfaces, as they lack support. Support is provided for in-plane orientation, while out-of-plane detection should be limited to ±45 degrees for optimal performance. The minimum size of code modules should be at least two-thirds of a pixel. Codes with higher versions have smaller modules, which affects their detectability. A trade-off characterizes the relationship between the distance and size of codes for optimal detection. The accompanying graph in Figure 1 illustrates this trade-off, which emphasizes the importance of balancing these elements to ensure a reliable code reading. While this paper initially discusses qr codes due to their versatility, error correction capabilities, and ability to store larger amounts of data, the implementation of 1D barcodes is chosen for practical reasons. In the healthcare sector, particularly with medical vials, 1D barcodes are the standard. As such, our implementation reflects real-world use cases where medical vials are predominantly labeled with 1D barcodes. Although qr codes offer additional benefits, they are not yet commonly adopted for vial labeling, making 1D barcodes a more suitable choice for our study.

### 1.2. Application Scenario

Addressing Real-World Challenges in Medical Emergencies and Integration of MR Technology

Our work specifically targets the challenges faced by paramedics and doctors in high-pressure emergency situations, where the accurate and rapid identification of medical vials is crucial. One of the most significant challenges is distinguishing between look-alike vials, which can lead to medication errors with potentially severe consequences. This focus on resolving a real-world problem in the medical field represents a significant advancement beyond generic barcode tracking systems. Medication dosages are determined by factors such as patient age, weight, height, and other relevant criteria. Determining the correct medication dosage is essential based on patient information, particularly in emergency situations when vials can look similar. Our approach integrates mixed reality (MR) technology using the Microsoft HoloLens 2, which allows for the real-time overlay of digital information onto the physical world. This integration is not just a straightforward implementation of barcode tracking but an innovative use of MR to enhance the accuracy and efficiency of vial identification in medical contexts. The use of HoloLens 2 for this purpose is a relatively new concept and represents a significant advancement over traditional methods, which rely solely on standard barcode scanners.

This study contributes to the field in the following ways:

- Integration of Mixed Reality (MR) with real-time barcode detection in medical settings.
- A hands-free interface using HoloLens 2, enabling faster and more efficient vial recognition.
- A hybrid approach that integrates a Bluetooth scanner with the MR device.
- Enhanced accuracy and robustness under varying environmental conditions (e.g., lighting and vial positioning).
- Our hybrid approach is an on-device execution approach that does not need any additional software or computer device attention, unlike the barcode-only method.

We perform our work in three phases. The first phase is our data collection and preprocessing phase. In this phase, we collect various real-time vials with barcodes of different sizes. The second phase is the detection phase. This phase includes the environment’s setup, the application’s development, and the integration of the database with the application. The third phase is the experiment part, where we implement our application, use it with real-time data, and obtain the result. In the following sections, we will discuss some related research work, related terminologies, methodology, results, and discussions, and end with conclusions and future work.

## 2. Related Work

Accurate vial identification is crucial for medical service providers since they have to choose the correct vials out of possibly hundreds of vials in their everyday operations. Machine learning has significantly enhanced object detection by employing several techniques, including classification [6], segmentation [7], and others. However, the primary focus is to ensure precision when utilizing machine learning approaches. Precise identification is crucial when dealing with medications that resemble each other. Text-based detection or barcode-based detection is particularly effective in solving such detection problems. Current approaches to vial recognition primarily focus on standalone barcode scanning systems or machine learning-based image recognition methods [8]. While effective in some cases, these methods struggle with maintaining accuracy in dynamic environments or under difficult lighting conditions due to the limited computational capabilities of MR devices [9]. For instance, standard barcode readers often fail when the barcode is distorted or not clearly visible [10] The use of 1d and 2d (qr code) barcode reading is widely common in various fields such as business, industry healthcare, etc. This is a very effective way to perform the job very efficiently and effectively [11,12]. In their discussion of qr code application in healthcare education, Chirag et al. [13] identified four recurring themes. Increasing participant engagement, simulating training, just-in-time (JIT) learning, and helping with administrative duties during training were a few of these. Most people see the deployment of codes favorably. Among the difficulties noted were those related to the technological infrastructure, the scarcity of smartphones, and the reluctance to utilize them in particular settings. However, although 1d and 2d barcode reading technology is fairly common in smartphone and laptop computer applications, barcode reading in a mixed reality setup is a new and demanding research topic among researchers of different backgrounds. Huo et al. [14] discussed a way of recognizing the qr code based on artificial intelligence. This article uses backpropagation neural networks to fix qr code distortion with an adaptive median filter approach. Combining the two results in a 14% increase in reading rate by fitting distorted code images into geometric deformation patterns. Although it shows great improvement, the recognition is limited to a few systems and setups. Medical vial detection using barcode or qr code detection is very common using smartphones or desktop computers. The use of mixed reality technology, such as the implementation of smart VR devices like Microsoft Hololens to complete patient assessment and medicine recommendations, is a new concept. One of the steps for such a VR application includes accurately identifying the vial to prescribe to the patient when some look similar. Agnieszka Florkowska et al. [15] conducted a methodological analysis on the detection of two commonly used marker kinds, namely, qr codes and ArUco markers, utilizing Hololens 2 and specialized algorithms specifically built for this purpose. The use of mixed reality technology such as Hololens 2 devices is increasing day by day [16]. Nassir Navab [17] discussed the use of a Hololens device for training the medical technologist, which shows promise because of the very engaging and interactive environment. Matko Šarić et al. [18] proposed a telemedicine system based on XR technology that generates a 3D representation of the patient using body position estimation, eliminating the need for complicated 3D body reconstruction. The system comprises an augmented reality (AR) client on Microsoft HoloLens 2 designed for local doctors, a virtual reality (VR) client on HTC Vive Pro intended for remote clinicians, and a backend server. Both parties engage the avatar and provide annotations on the 3D model. WebRTC guarantees optimal frame rates and minimal latency. In this study, we propose a novel solution that leverages Mixed Reality (MR) technology through the HoloLens 2 platform. Our approach integrates real-time barcode detection and vial recognition, allowing healthcare professionals to operate hands-free while receiving instant feedback in an augmented environment. By combining MR with advanced image processing techniques, our system overcomes the limitations of traditional barcode scanning and enhances operational efficiency and accuracy.

As seen in Table 1, our proposed method using Mixed Reality on the HoloLens 2 outperforms the traditional barcode approach in terms of real-time performance and accuracy. Although ML methods show promising results and accuracy, due to the limited computational configuration of the MR device, their result is slow and sloppy. The ability to detect vials under dynamic and challenging conditions sets our method apart, making it suitable for critical healthcare environments.

## 3. Related Terminologies

### 3.1. MRTK(Mixed Reality Tool Kit)

Mixed reality is a technology that integrates augmented reality (AR) and virtual reality (VR) to create immersive experiences that blend the digital and physical worlds. Microsoft HoloLens, a prominent mixed reality (MR) technology, has been utilized in several healthcare applications, ranging from surgical preparation to medical instruction. The hands-free operation and spatial awareness features of this technology offer novel possibilities for identifying medical vials. A study was conducted to evaluate the educational efficacy of mixed reality (MR) technology in medical education. The study participants included 211 students and 47 faculty members from a medical college. A study revealed that 70% of the students and 60% of the faculty perceive MR-enhanced teaching as more beneficial than traditional approaches, with a particular emphasis on the use of 3D visualization in anatomy lessons. However, the accessibility of MR technology was restricted, as only 5% of students and 17% of professors used it regularly [20].

### 3.2. Barcode Detection

Two-dimensional barcode detection is very common and has already been established using mixed reality technology. However, 1D code detection, such as barcode detection, still faces a lot of challenges due to its size and the limitations of devices. As we work with the specific kind of barcode detection, we focus on reading the barcode using Microsoft Hololens 2.

### 3.3. MR Technology and Microsoft Hololens 2

According to Milgram and Kishino (1994) [21], mixed reality encompasses a spectrum of environments that merge real and virtual worlds, allowing physical and digital objects to coexist and interact in real-time. The Microsoft HoloLens 2 is a notable development in mixed reality technology, with strong features for a variety of uses, especially in medical applications. Its real-time integration of digital and physical surroundings offers useful tools for patient care, surgical planning, medical education, and remote help. HoloLens 2 has the potential to completely change how medical professionals engage in digital information in their practices despite still facing some implementation challenges. Our work leverages the Microsoft HoloLens 2, a state-of-the-art mixed reality device, to overlay digital information onto the physical world of healthcare professionals. In our system, users can view and interact with digital representations of vital information, which are superimposed directly onto the physical vials themselves. The HoloLens 2 provides visual augmentation and real-time interaction with these digital elements, such as selecting, manipulating, and verifying vial information through gesture and voice commands. This interactive component further substantiates our implementation as an MR application, as users are simultaneously engaging with the real world (the physical vials) and the digital overlays (barcode information and vial details). Additionally, our application demonstrates the potential of MR to improve decision-making processes in high-stress environments by providing immediate access to critical information without disrupting the user’s interaction with the physical world. Given that medical data are highly sensitive, Microsoft ensures that HoloLens 2 follows strict data security measures. The device incorporates encryption and compliance with healthcare standards like HIPAA (Health Insurance Portability and Accountability Act), protecting patient information and ensuring that any scanned data remain secure.

## 4. Methodology

**Figure 2.**
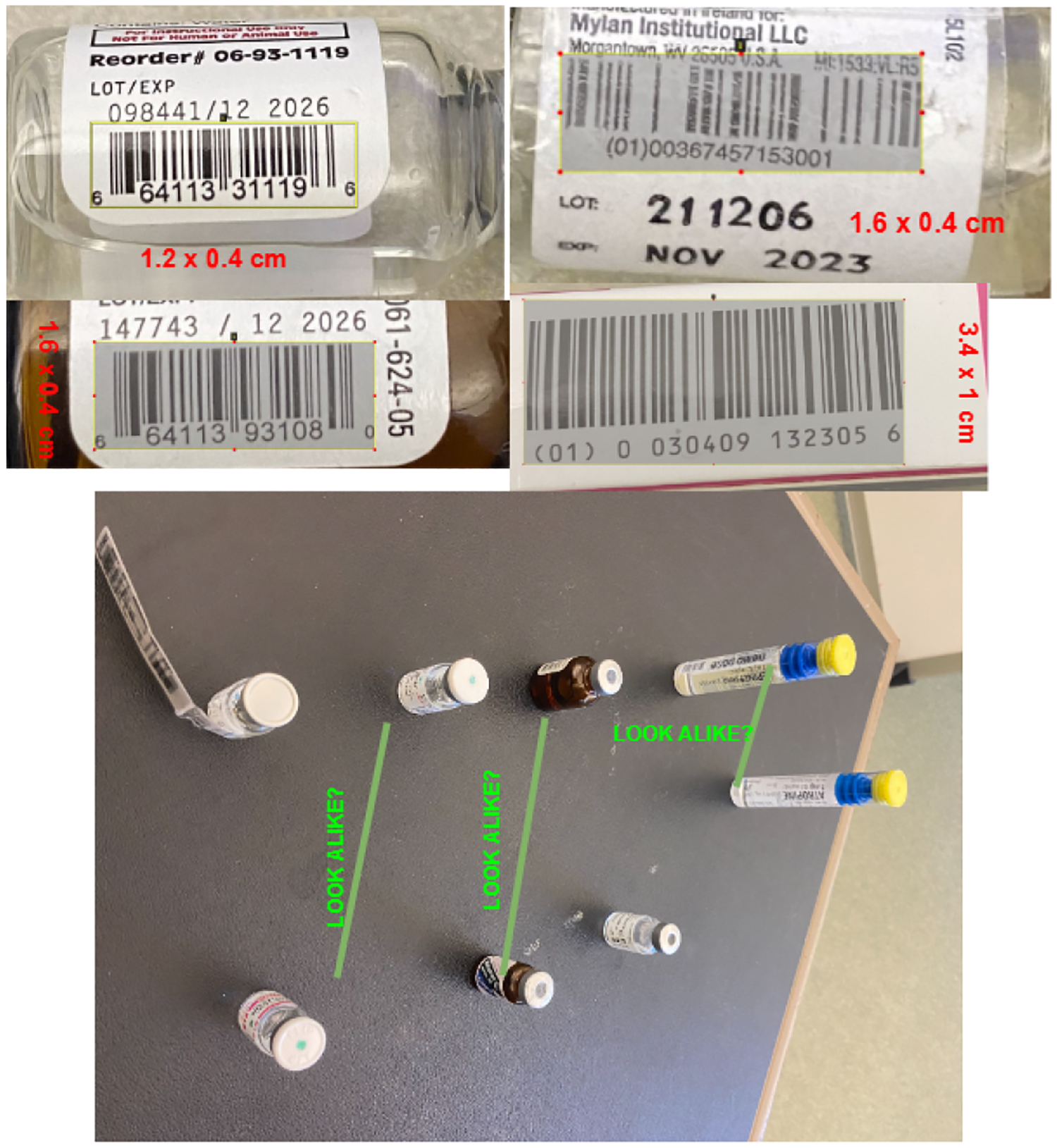
Represents the sample vial images with barcodes of different sizes and vials with a similar look.

The proposed barcode detection technique was implemented on the Microsoft HoloLens 2 by developing an application using the Unity platform, integrated with the Mixed Reality Toolkit (MRTK) to harness HoloLens’ mixed reality capabilities. The HoloLens 2 camera was configured to capture real-time video at a resolution of 1920 × 1080 and 1280 × 720 pixels at 30 frames per second, enabling high-quality image acquisition. The video feed was processed frame by frame using the modified ZXing library for barcode decoding. To improve detection accuracy, the image preprocessing technique Gaussian blur was applied to reduce noise, while Sobel edge detection highlighted key features like barcode edges. In cases where smaller barcodes could not be detected by the HoloLens camera, an external Bluetooth barcode scanner was incorporated to ensure successful detection. The barcode data were decoded using the Reed–Solomon algorithm, which corrected potential errors and ensured high precision. The decoded information was then displayed in real-time on the HoloLens 2 interface using TextMeshPro for clear text rendering, and barcode-related vial information was retrieved from a connected SQL server to provide the user with additional data. This real-time system was designed to enhance the accuracy and efficiency of vial detection in mixed reality environments, crucial for emergency medical scenarios. In our implementation, 1D barcodes were selected over qr codes due to their widespread use in medical environments. Medical vials in real-world settings are typically labeled with 1D barcodes, and our system was designed to work seamlessly with the existing infrastructure. While QR codes offer greater data storage and error correction capabilities, the specific use case for vial detection, as shown in Figure 2, necessitated using 1D barcodes for compatibility and practical purposes. Our methodology consists of data collection and preprocessing, setting up the environment and implementation, scanning barcodes, communicating with the server, and theoretical foundations. In the following, we discuss each part separately.

### 4.1. Data Collection and Preprocessing

For our experiment, we collected various raw medical vials with barcodes of different sizes. We measured the size of the barcode for our experiments. We utilized ImageJ software for the measurement. After that, for the implementation of our experiment, we achieved the data through the continuous video stream provided by the HoloLens 2 camera. The WebCamTexture was initialized with a fixed resolution of camera pixels and a frame rate. This setup captured real-time video of the user’s environment, specifically targeting barcodes visible in the frame. Each frame from the camera feed was converted into a pixel color array (Color32[]), which was the input for barcode detection. The video stream allowed for continuous and dynamic scanning of barcodes in real-world conditions, such as varying distances, angles, and lighting. The camera display object was used to display the camera feed on the screen, simulating a real-time feedback loop during the scanning process. The processing pipeline began with the extraction of pixel data from each frame in the video stream. These pixel data were fed into the ZXing barcode decoding library, which attempted to identify and decode the barcode present in the current frame.

### 4.2. Preparing the Environment

We used the Unity platform to develop the application for HoloLens. In our experiment, we developed the application for Hololens 2. Hololens 2 has overcome various limitations of Hololens 1, such as better autofocus in the camera setting. In our experiments with the HoloLens 2, we systematically varied both the lighting and detection distances to evaluate barcode recognition performance. To simulate real-world emergency environments, two lighting conditions were tested—normal indoor lighting (300 lux) and low light (50 lux). Detection distances ranged from 15 cm to 30 cm, with measurements taken at regular intervals. The HoloLens 2’s autofocus and auto-exposure features adapted to these changes, ensuring consistent image capture quality. We used a 1920 × 1080 resolution at 30 fps to balance image quality and performance, particularly for detecting small barcodes. Our experiment included both hardware and software requirements. The hardware component consisted of a Microsoft Hololens device, and to develop the software, we used the Unity platform. We created our project with Unity UWP version 2022.3.1f1 for our experiment. After opening the unity-specific version, we installed the MRTK toolkit in the project to utilize the mixed reality features. Table 2 and Figure 2 demonstrate the vial information and visualization of the code that we used for our experiment.

### 4.3. Implementation

**Figure 3.**
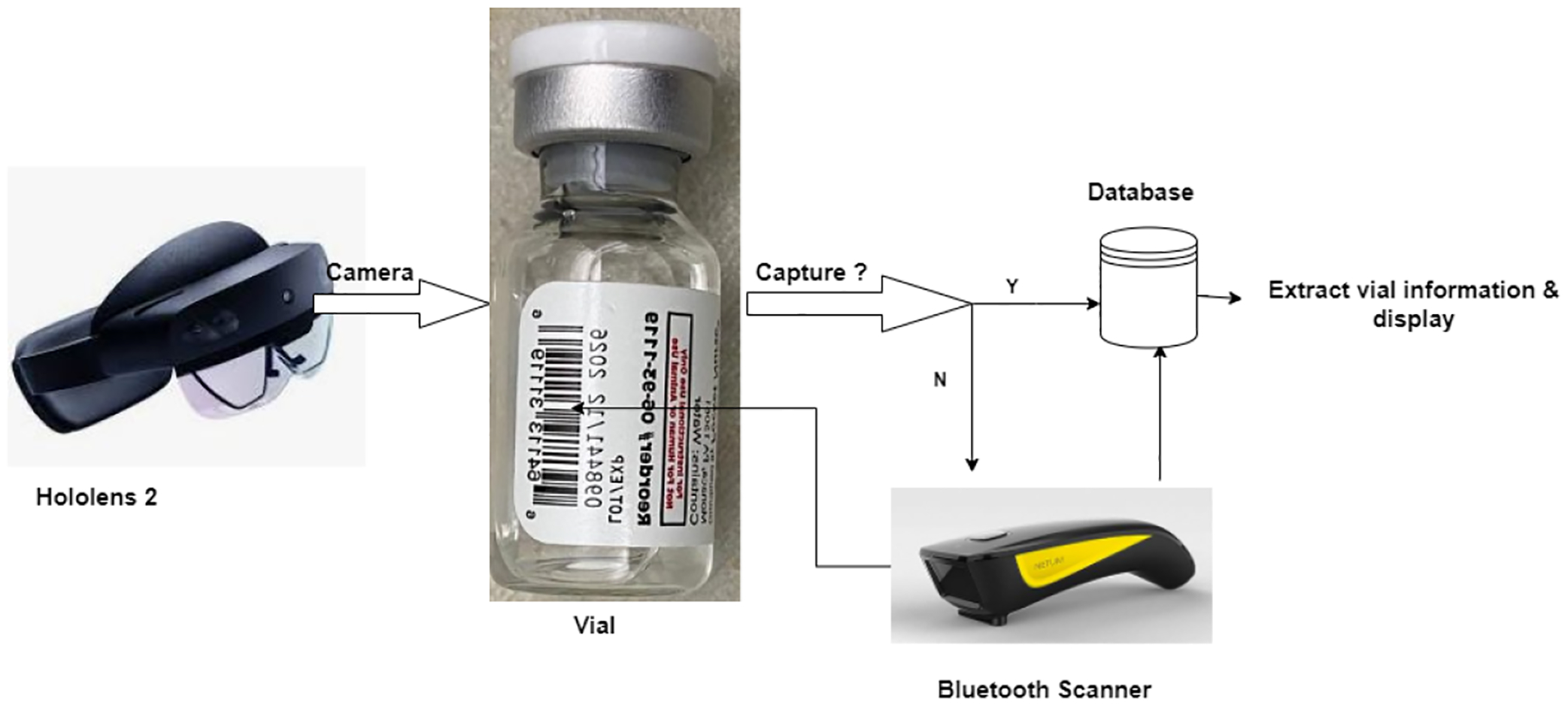
The step-by-step process of the code scanning. Hololens 2 camera captures the barcode of the vial. If it is unable to do so, then the scanner attached will get the code and request its database to get related information.

**Figure 4.**
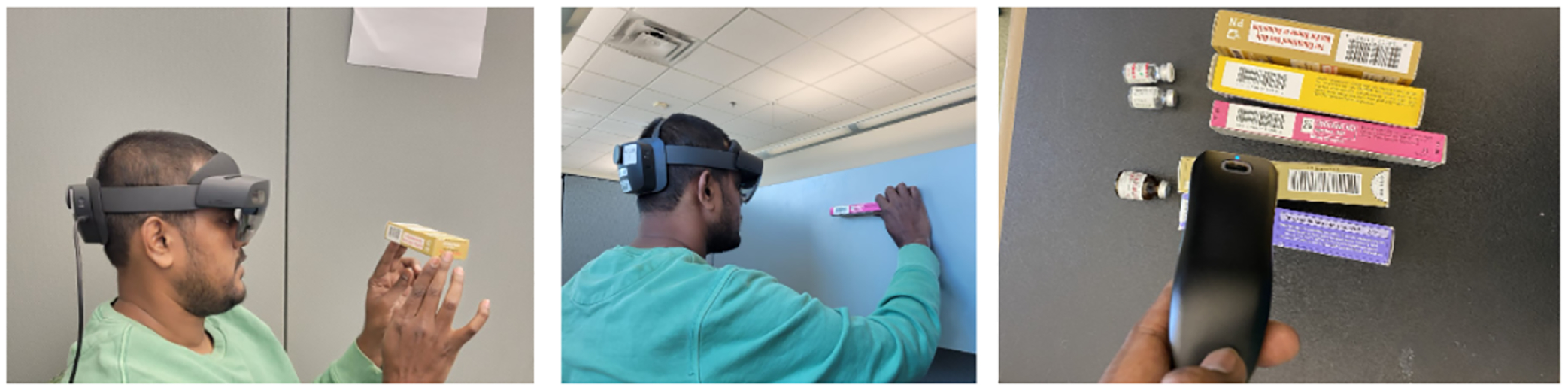
The image represents a user scanning a barcode on a small package using HoloLens 2, a user scanning a barcode on a whiteboard with HoloLens 2, and a barcode scanner with various vials used for testing.

The application was developed using Unity, utilizing the WebCamTexture class for camera access, modified ZXing library for barcode decoding, and UnityWebRequest for server connectivity. The TextMeshPro interface was created to achieve improved text rendering. Barcode scanning is pretty common and well developed in different use cases. However, there are several limitations to implementing HoloLens 2 for mixed reality applications. To scan the vial code, first utilize the Hololens 2 camera to scan the code displayed on the vial. However, it is noticeable that because of specific hardware limitations, the HoloLens 2 camera cannot scan the code of small sizes, which is crucial for vial detection. We introduced a new hybrid approach to scanning the code, which involves using a Bluetooth barcode reader and HoloLens 2. If the Hololens 2 camera cannot detect the vial and the code, the scanner attached to it will try scanning the code, which we found successful in this setup. Figure 3 represents the step-by-step process of our experiment. Figure 4 demonstrates the barcode detection process using HoloLens 2 and the modified ZXing library for different types of packages. In the first image, the user scans a barcode on a small package using HoloLens 2, showcasing its ability to detect barcodes on compact objects. In the second image, the user scans a barcode placed on a whiteboard, highlighting the versatility of the HoloLens 2 in detecting barcodes on flat surfaces. Finally, the last image shows a barcode scanner alongside various vials used for testing, further emphasizing the range of objects and scenarios considered during the barcode detection evaluation.

### 4.4. Barcode Scanning

The application starts the webcam by setting its resolution to 1920 × 1080 at a frame rate of 30 frames per second. It saves the width and height of the camera feed for future reference when decoding barcodes. The text fields used to show the results, namely, ResultText and DatabaseResultText are configured using TextMeshProUGUI components. A new instance of the IBarcodeReader interface is created using the ZXing library. This instance is configured with particular decoding parameters that disable the AutoRotate and TryHarder features. This configuration is performed to enhance performance and accelerate the scanning process. A coroutine named ScanBarcode() starts to handle the camera data consistently. This involves turning the feed into a Color32 array, which is then sent to the barcode reader for decoding. Once a barcode is decoded correctly, its value is shown in the ResultText field and transmitted to the server for additional processing. In addition, the application actively receives keyboard input from Bluetooth scanners to form a barcode string. Once the string is fully formed, it undergoes a similar processing procedure.

### 4.5. Server Communication

After successfully scanning a barcode, the program establishes communication with a server in order to retrieve relevant information that is stored in an SQL database. Communication is enabled via an HTTP GET request that is formed within the GetDataFromServer coroutine. This request includes the scanned barcode as a query parameter in the URL. The application uses the UnityWebRequest class to send this request and awaits the server response. Upon a successful request, the server will provide a JSON response that includes the pertinent data. The JSON answer is subsequently deserialized into an array of Data objects utilizing a helper class. Data that have been converted from a serialized format containing information such as barcode, name, dose, and further details are shown in the DatabaseResultText field. The server-side script handles the incoming GET request by extracting the barcode, querying the SQL database, formatting the query results as a JSON array, and returning this array back to the application as the HTTP response, thus concluding the data retrieval process.

**Figure 5.**
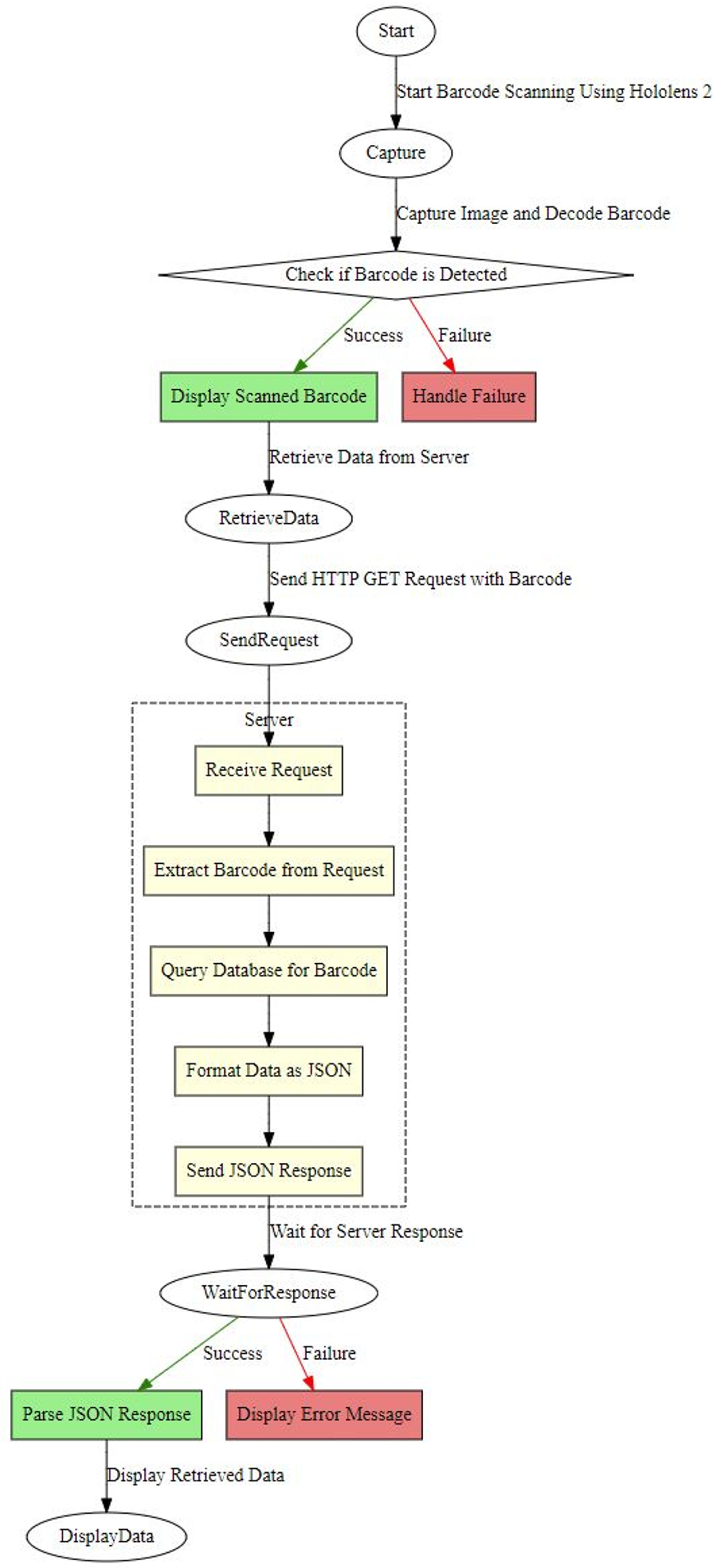
Flowchart of the vial detection process using the mixed reality technology.

The flowchart in Figure 5 illustrates a procedure in which a Unity program employs barcode scanning to capture and decipher images from a camera texture. After correctly identifying a barcode, it shows the scanned code and starts a procedure to retrieve the data. This process entails transmitting an HTTP GET request to a server, which then receives and extracts the barcode information. The server subsequently executes a database query using SQL to retrieve the relevant data, convert them into JSON format, and transmit the response back to the Unity application. The application refreshes the user interface with the obtained data if the server’s response is successful or shows an error message if the retrieval fails. This approach guarantees optimal communication between the Unity frontend and the backend server for smooth data management and presentation.

### 4.6. Theoretical Foundations

#### 4.6.1. Pinhole Camera Model

The pinhole camera model is a key concept in imaging that streamlines the process of capturing and presenting images. This approach involves the passage of light from a scene via a small opening, known as the pinhole, which then projects an inverted image onto a flat surface called the image plane. Although the model is simple, it offers a fundamental understanding of perspective projection and is extensively utilized in the fields of optics and computer vision. The pinhole camera model describes the relationship between 3D points in the world and their 2D projections on the image plane using a linear transformation. The geometric representation of the pinhole camera model can be described by the following equation:

(1) $$s\cdot \left[\begin{array}{l} u\\ v\\ 1 \end{array}\right]=\left[\begin{array}{ccc} f_{x} & 0 & c_{x}\\ 0 & f_{y} & c_{y}\\ 0 & 0 & 1 \end{array}\right]\cdot \left[\begin{array}{l} X\\ Y\\ Z \end{array}\right]$$

where we have the following:

- (u,v) are the pixel coordinates on the image plane;
- (X,Y,Z) are the coordinates of a point in 3D space;
- $f_{x}$ and $f_{y}$ are the focal lengths along the x and y axes;
- $c_{x}$ and $c_{y}$ are the principal point offsets;
- s is a scaling factor.

The HoloLens 2 has the following regular values: focal lengths of around 1390 pixels for both the x and y axes $\left(f_{x}\approx 1390,f_{y}\approx 1390\right)$, primary point offsets of approximately 1024 pixels for the x-axis and 540 pixels for the y-axis $\left(c_{x}\approx 1024,c_{y}\approx 540\right)$, and a scaling factor of 1 (s=1). These characteristics played a vital role in effectively calibrating the imaging apparatus and guaranteeing accurate barcode measurements in our studies.

#### 4.6.2. Image Processing Techniques

Image preprocessing techniques are crucial for preparing captured images to ensure accurate barcode detection. These strategies are designed to improve the quality of the images, minimize noise, and emphasize the important elements necessary to accurately identify barcodes. Two often employed methodologies in image processing are noise reduction and edge identification, which are executed by algorithms such as Sobel or Canny.

For noise reduction, we implemented the Gaussian blur techniques in the script, which is important for removing random variations in pixel intensity that can obscure important details in an image.

The image is smoothed using a Gaussian kernel, which involves averaging pixel values and minimizing high-frequency noise:

(2) $$I_{\text{blur}}(x,y)=\sum_{i=-k}^{k}\sum_{j=-k}^{j}I(x+i,y+j)\cdot G(i,j)$$

where G(i,j) is the Gaussian kernel and k is the kernel size. We adopted a Gaussian kernel of dimensions 5 × 5 to reduce noise. The choice of this kernel size was made to strike a balance between efficiently reducing noise in the image and retaining crucial information necessary for precise barcode identification.

For edge detection, we implemented the Sobel edge detection techniques, which is a critical step in image processing that helps identify the boundaries of objects within an image. The Sobel operator calculates the gradient of the image’s intensity at every pixel, highlighting areas with high spatial frequency that represent edges.

The Sobel operator uses two 3 × 3 kernels, one for the horizontal gradient $\left(G_{x}\right)$ and one for the vertical gradient $\left(G_{y}\right)$:

(3) $$G_{x}=\left[\begin{array}{lll} -1 & 0 & +1\\ -2 & 0 & +2\\ -1 & 0 & +1 \end{array}\right],G_{y}=\left[\begin{array}{ccc} -1 & -2 & -1\\ 0 & 0 & 0\\ +1 & +2 & +1 \end{array}\right]$$

The magnitude of the gradient at each pixel is then given by

(4) $$I_{\text{edges}}(x,y)=\sqrt{G_{x}(x,y{)}^{2}+G_{y}(x,y{)}^{2}}$$

where $G_{x}(x,y)$ and $G_{y}(x,y)$ are the gradients in the x and y directions, respectively. In our specific application, we decided on the Sobel edge detection approach instead of the Canny edge detector due to its simplicity and computing efficiency. The Sobel operator computes the gradients in the horizontal and vertical directions, which is effective in detecting the edges of barcodes that are often aligned horizontally or vertically. The Sobel operator is used mainly to detect edges that are horizontal or vertical. However, it is also capable of capturing diagonal lines by considering the gradients in both directions. The Canny edge detector provides enhanced edge detection capabilities, particularly for diagonal lines. However, its computational complexity has increased due to the inclusion of extra processes such as non-maximum suppression and hysteresis thresholding. During our preliminary examinations, we found that the Sobel operator yielded sufficient results for the barcode images taken with HoloLens 2.

#### 4.6.3. Barcode Decoding

For barcode decoding, we implemented the Reed–Solomon algorithm. The Reed–Solomon algorithm is crucial for deciphering barcodes, particularly in situations where errors may arise from noise or distortion. This algorithm corrects mistakes and guarantees the precision and dependability of the decoded data. Reed–Solomon codes are denoted as RS(n,k), where n is the total number of code symbols and k is the number of data symbols. The encoding process involves creating a message polynomial m(x) of degree k-1, and a generator polynomial g(x) of degree n-k. The codeword polynomial c(x) is obtained by multiplying m(x) by $x^{n-k}$ and adding the remainder r(x) from the division by g(x):

(5) $$c(x)=m(x)\cdot x^{n-k}+r(x)$$

when a barcode is scanned, the received polynomial r(x) may contain errors:

(6) r(x)=c(x)+e(x)

where e(x) represents the polynomial of errors. Syndromes $S_{i}$ are calculated to detect errors:

(7) $$S_{i}=r\left(\alpha^{i}\right)$$

for i=0,1,…,n-k-1. The error locator polynomial σ(x) and the error evaluator polynomial ω(x) are used to find and correct errors, ensuring reliable decoding of the barcode data.

#### 4.6.4. Performance Metrics: Frame Rate

The frame rate measures the system’s operational speed in processing barcode detection and rendering augmented reality elements. It is defined as the number of frames processed per second (fps) and is given by the reciprocal of the average processing time per frame:

(8) $$\text{Frame}\;\text{Rate}=\frac{1}{\text{Average}\;\text{Processing}\;\text{Time}\;\text{per}\;\text{Frame}}$$

A higher frame rate indicates a more responsive and efficient system, which is crucial for real-time applications. In the context of barcode detection and augmented reality on the HoloLens, maintaining a high frame rate ensures that barcodes are detected and processed quickly and that AR elements are rendered smoothly, providing a seamless user experience.

## 5. Result and Discussion

**Figure 6.**
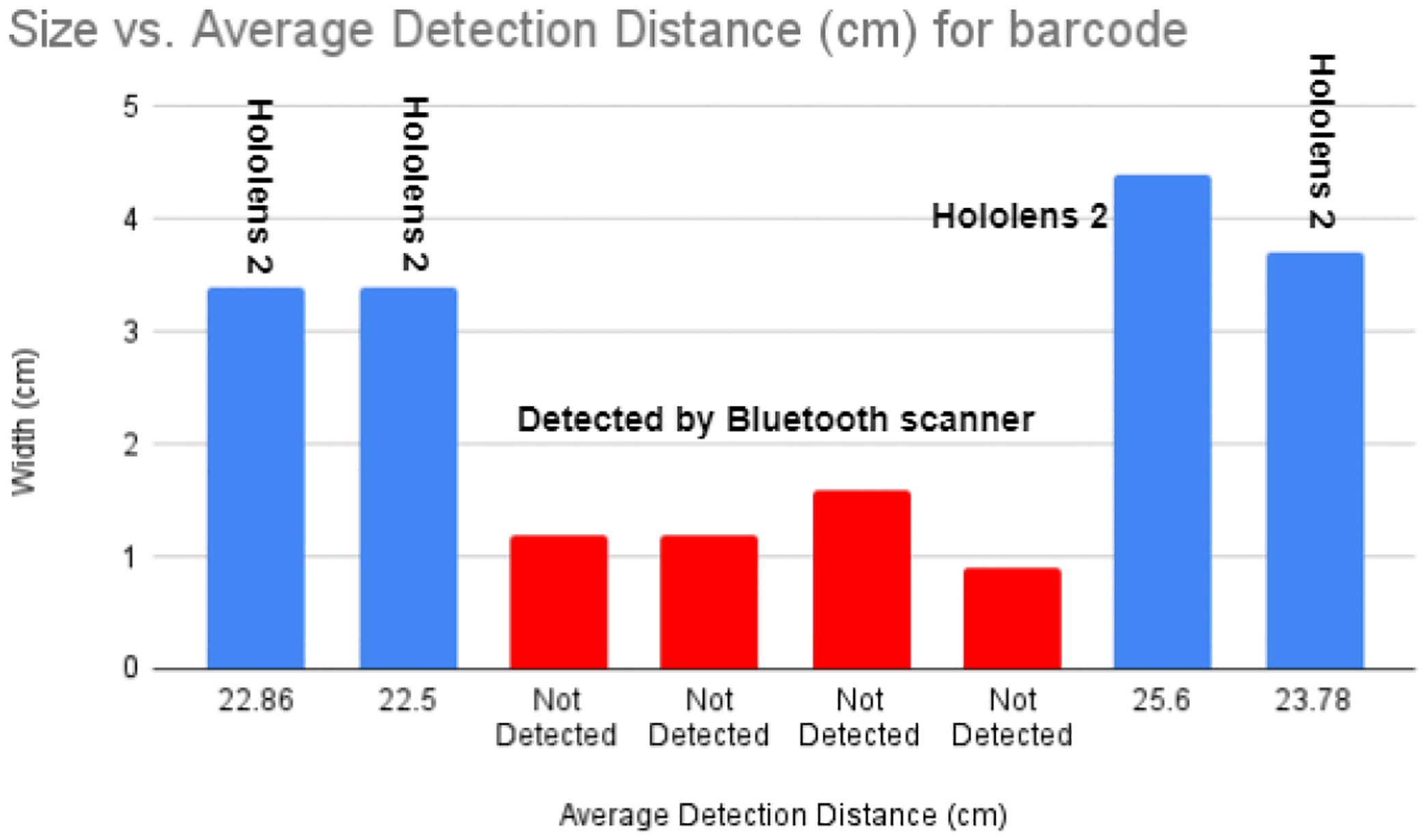
Represents the results of barcode size and detection distance. Blue color samples were detected by the MR device only. The red one was detected by an assisted Bluetooth scanner, which attached to the MR device.

Table 3 presents a comprehensive summary of barcode detection capabilities for different medications using the HoloLens 2 camera and an external scanner. The HoloLens 2 camera and the external scanner consistently detected larger barcodes, such as lidocaine HCI (3.4 × 1 cm) and epinephrine (4.4 × 1.3 cm), at distances of around 22–30 cm. The barcode processing duration, covering frame capture and decoding, varied for each vial, ranging from 50 to 70 milliseconds. Information retrieval from the database required additional time due to the establishment of a server connection and the subsequent data retrieval process, which ranged from 300 to 315 ms. However, the HoloLens 2 camera did not recognize tiny barcodes such as amiodron (1.2 × 0.5 cm) and fentanyl citrate (0.9 × 0.4 cm), but they were correctly identified by the external scanner. This discrepancy emphasizes the camera’s constraints in identifying smaller barcode sizes and emphasizes the greater sensitivity of the external scanner for different barcode dimensions. The table highlights the significance of enhancing detection algorithms and potentially including better-resolution cameras to enhance the HoloLens 2’s ability to recognize barcodes, especially for smaller or more intricate barcode formats in medical environments. The data presented in the table below were collected under normal lighting conditions with the camera resolution set to 1920 × 1080. Figure 6 shows the trade-off between the barcode size and detection distance. The combination of the barcode-based method and MR device gives the optimal results as well as hands-free detection in dynamic conditions.

**Figure 7.**
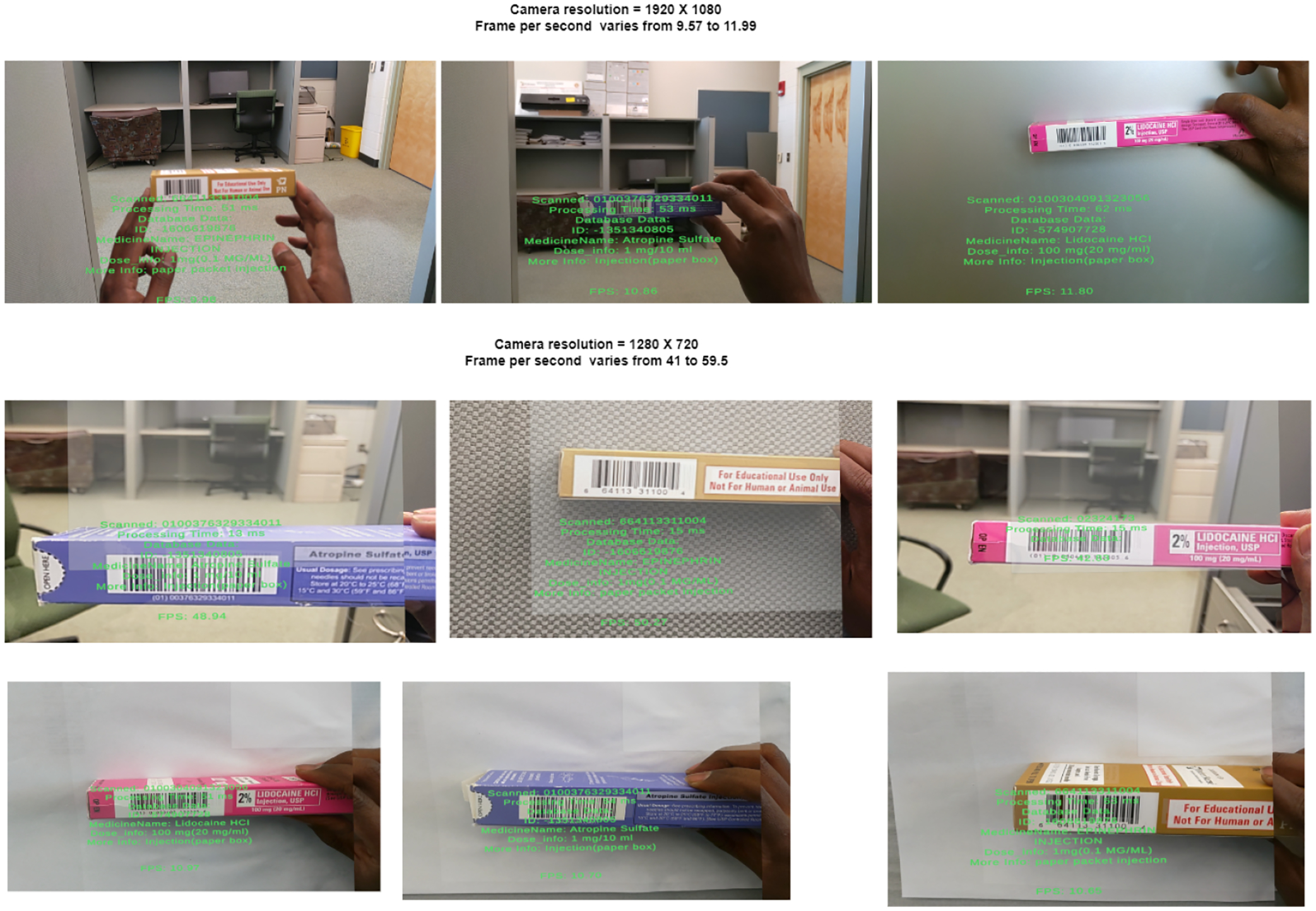
Represents the results after scanning the barcode. Scanning time varies for the Hololens camera based on the barcode size and camera resolution.

Figure 7 shows the result we obtained after scanning the barcode. In row 1, we configure our webcam resolution to 1920 × 1080, which is the maximum video resolution for the HoloLens 2 camera. In this configuration, the frames per second (FPS) range from 9.57 to 11.99. The camera resolution in the second row is configured at 1280 × 720, with the frames per second ranging from 41 to 59.5. The presence of a white background significantly enhances detection frequency, thus demonstrating that lighting conditions and background are crucial factors in the effective identification of the code. The scanned code is sent to the database, and the vial information is pulled from the database based on the related barcode. The frame rate is a vital performance measure for assessing the effectiveness and speed of our barcode identification and augmented reality (AR) system on the HoloLens 2. The frame rate data were obtained by utilizing a FrameRate Calculator that was incorporated into our Unity program. This calculator measured the duration it took to process each frame under various test circumstances. The frame rate was assessed across different scenarios, encompassing standard lighting settings, dim lighting circumstances, and locations with a high density of barcodes. Every scenario underwent a 1 min testing period to guarantee a thorough assessment. The following Table 4 summarizes the average, maximum, and minimum frame rates observed during our tests.

**Figure 8.**
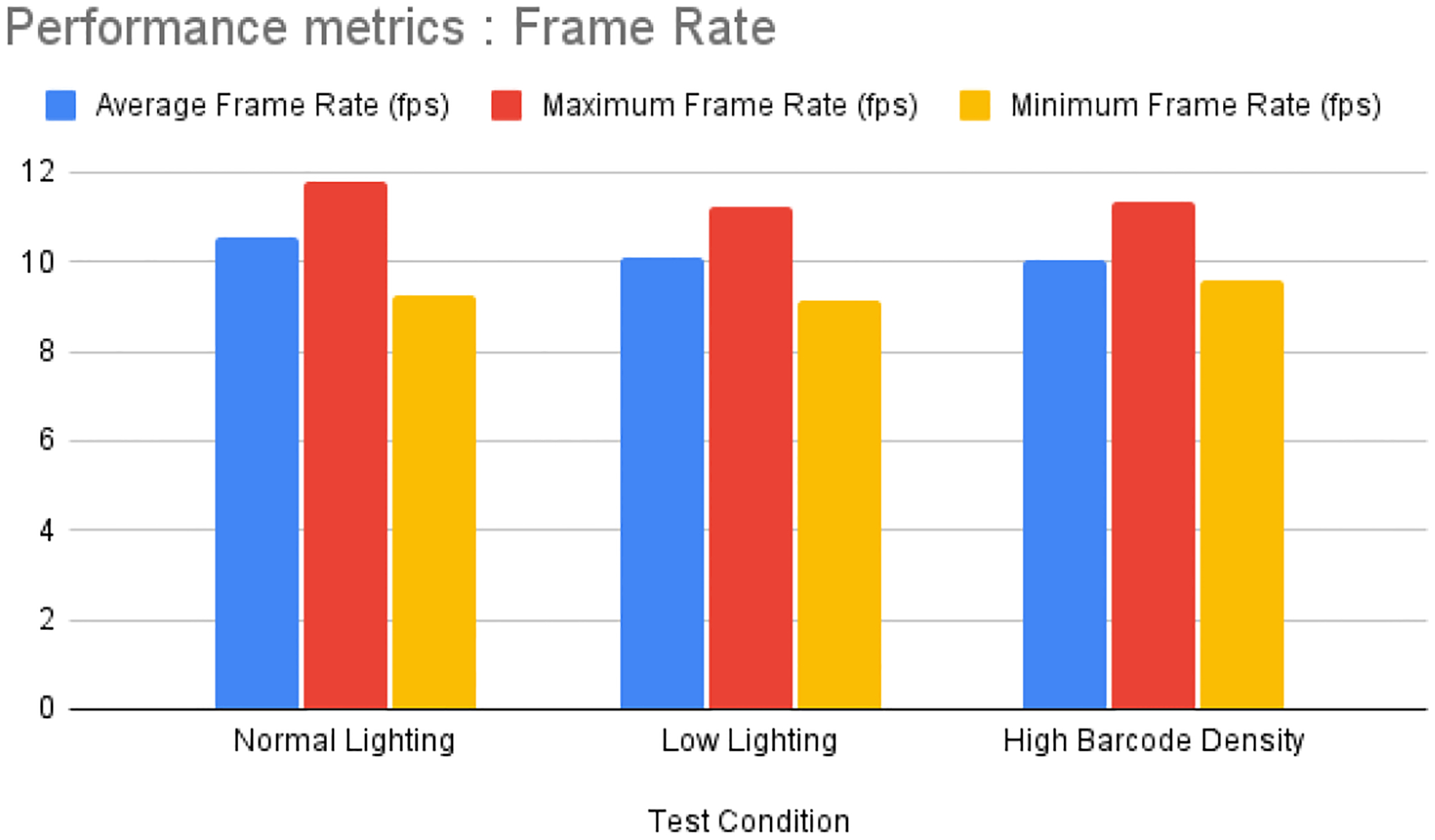
Represents the visualization of frame rate in different lighting setup for Hololens 2 camera.

Table 4 delineates the average, maximum, and minimum frame rates recorded during our experiments across various situations. Figure 8 depicts the frame rate performance under different conditions. In standard lighting conditions, the system attained an average frame rate of 10.54 frames per second (fps), with a peak frame rate of 11.80 fps and a lowest rate of 9.26 fps. This suggests that the technology operates effectively under standard illumination conditions. Under low lighting conditions, the average frame rate fell to 10.10 fps, with a maximum of 11.22 fps and a lowest of 9.13 fps. The decline in average frame rate indicates that the camera’s performance is adversely impacted by diminished lighting, perhaps owing to the heightened computing demands for picture enhancement in low-light conditions. The most substantial effect on the frame rate was noted in situations with an elevated barcode density. The average frame rate decreased to 10.05 fps, with a maximum of 11.33 fps and a minimum of 9.57 fps. The reduction in average frame rate under high barcode density settings indicates the heightened processing demand of managing many barcodes concurrently. The results indicate that the system sustains a relatively high frame rate in normal illumination, but its performance diminishes marginally in low lighting and more substantially under conditions of high barcode density. To enhance real-time performance, additional optimizations may be required, especially in augmenting picture preprocessing for low-light environments and refining the barcode detection algorithm for high-density situations.

The following table summarizes the detection accuracy, average processing time, and frame rate observed at each resolution.

**Figure 9.**
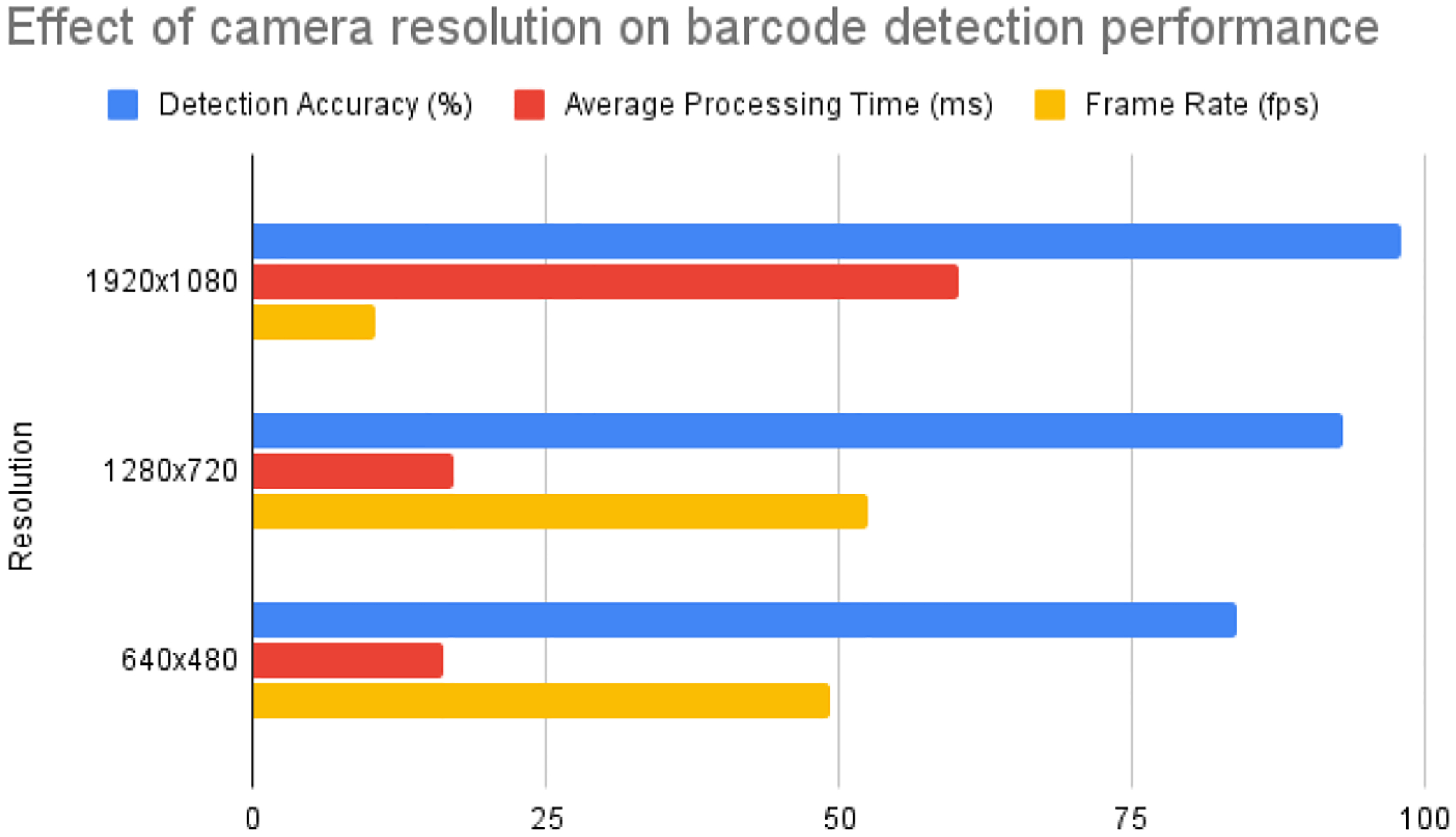
Represents the visualization of detection accuracy for different camera resolution of Hololens 2.

Table 5 indicates that the best detection accuracy of 98% was achieved using a high resolution of 1920 × 1080. This resolution ensured that barcodes were detected with few errors for the indicated sizes. However, this resolution led to the highest processing time of 71 milliseconds and a frame rate of 11.80 frames per second. The medium resolution (1280 × 720) obtained a detection accuracy of 93%, with a processing time of 21 ms and a frame rate of 59 fps. The detection accuracy was the lowest at 84% for low resolution (640 × 480) but had the fastest processing time of 16.2 ms and the highest frame rate of 49.3 fps. Figure 9 presents the visualization of the detection accuracy for different resolution configurations.

Our findings suggest that the use of high resolution greatly enhances the precision of detection by capturing highly detailed images. Ensuring reliable barcode detection is of the utmost importance, particularly in situations where the quality of the barcodes may differ. Nevertheless, the extended processing duration at higher resolution implies that the system needs to exert more effort in handling the intricate images, potentially affecting its real-time performance. Medium resolution provides an optimal trade-off between the precision of detection and the processing efficiency. Boasting a detection accuracy of 93% and a frame rate of 52.5 fps, this system delivers a commendable degree of performance for real-time applications while maintaining image quality without any notable compromise. Although low resolution has the advantage of faster processing time and higher frame rate, it sacrifices detection accuracy. This trade-off may not be suitable for applications that demand great accuracy in barcode detection, but it could be advantageous in situations where speed is more crucial than precision. Ensuring the ideal camera resolution is crucial to achieving a trade-off between the detection accuracy, processing time, and frame rate. Our research indicates that opting for a medium resolution of 1280 × 720 is the most pragmatic decision for real-time apps on the HoloLens 2. This choice strikes a balance between performance and image quality.

Maximizing the camera resolution is essential for improving the efficiency of barcode detection and augmented reality rendering in real-time applications. The significance of choosing a suitable resolution to achieve a balance between detection accuracy, processing efficiency, and user experience is illustrated in our study.

Our study aimed to explore the application of mixed reality (MR) technology, specifically using the HoloLens 2, to address the critical challenge of identifying look-alike medical vials:

- Our hybrid approach integrates the Bluetooth scanner with an MR device, which overcomes the limitations of both approaches while implementing it alone. Moreover, the approach still utilizes the maximum MR device capabilities such as hands-free detection and no additional software uses, unlike the barcode-based method.
- The integration of MR through HoloLens 2 allows healthcare professionals to overlay digital information directly onto the physical environment, providing an intuitive and interactive way to identify and distinguish between look-alike vials. This capability is especially beneficial in high-stress, time-sensitive medical scenarios, where quick and accurate identification is crucial.
- By using HoloLens 2, users can interact with the virtual representation of vials in real-time, making it easier to ensure that the correct vial is selected. This enhanced interaction is a significant advancement over traditional barcode scanning, which lacks the spatial awareness and contextual information provided by MR.
- Our experiments showed that the MR system, leveraging the HoloLens 2, could maintain high detection accuracy and speed even when dealing with look-alike vials in various environmental conditions. This includes challenging scenarios such as poor lighting or the presence of multiple vials that look similar. The system’s ability to provide real-time feedback and visual cues directly in the user’s field of view ensures that the right vial is identified quickly and accurately.
- The use of HoloLens 2 for vial detection is not limited to emergency situations. The underlying technology and approach can be extended to broader applications within healthcare and other industries. For instance, the system can be integrated into broader medical inventory management systems, assisting in everything from routine medication administration to more complex tasks like preparing for surgeries where correct vial identification is critical.
- The flexibility of MR technology also allows for the integration of additional data sources and functionalities, such as linking vial information to patient records, providing dosage instructions, or alerting users to potential medication interactions. This level of integration and functionality is what sets our approach apart, enabling a more holistic and efficient workflow for healthcare professionals.
- The results from our study demonstrate a significant reduction in vial identification errors, thus directly contributing to patient safety and compliance with medical ethical standards. By reducing the likelihood of misidentification through automated MR-enabled detection, our system provides a layer of security that is critical in environments where human lives are at stake.

While the HoloLens 2 comes with a higher cost and power considerations compared to traditional systems, its real-time processing, hands-free operation, and ability to ensure precise vial detection under various conditions make it an invaluable tool in medical settings. Additionally, robust security measures such as encryption, edge processing, and compliance with healthcare regulations ensure that the use of MR technology aligns with medical ethics and data security standards. The trade-off between cost and the increased safety and efficiency it brings to vial detection makes HoloLens 2 a justifiable choice in healthcare applications. Our HoloLens-based vial identification system demonstrated a precision rate of up to 98% under controlled conditions, significantly reducing errors compared to traditional human recognition. Studies have shown that in high-stress, fast-paced medical settings, human error rates in medicine identification can range from 5% to 15%, influenced by fatigue, cognitive overload, and visual similarity among vials. By contrast, the HoloLens system consistently maintains high accuracy without susceptibility to fatigue or external stressors

## 6. Conclusions and Future Work

This study investigated a novel method for detecting medicine vials using HoloLens 2 mixed reality technology with Bluetooth scanner. The findings of our study revealed that the HoloLens 2 camera encountered difficulties in accurately identifying barcodes on smaller vials. As an illustration, it effectively identified larger barcodes such as lidocaine HCI (3.4 × 1 cm) and epinephrine (4.4 × 1.3 cm) when they were, on average, 22.86 cm away. The processing time, which included frame capturing and decoding, was 57.6 ms. However, the device was unable to recognize smaller barcodes, such as amiodron (1.2 × 0.5 cm) and fentanyl citrate (0.9 × 0.4 cm), although an external barcode scanner accurately identified these. The investigation we conducted revealed a trade-off between camera resolution, detection accuracy, and processing time. The HoloLens 2, operating at a resolution of 1920 × 1080, demonstrated a remarkable detection accuracy of 98%. However, this achievement came at the cost of a greater processing time of 60.3 milliseconds and a frame rate of 10.54 frames per second. The resolution of 1280 × 720, which is considered medium, demonstrated a well-balanced performance with an accuracy rate of 93%, a processing time of 17.1 milliseconds, and a frame rate of 52.5 frames per second. In contrast, the low resolution of 640 × 480, although it offered the fastest processing time of 16.2 ms and a high frame rate of 49.3 fps, led to decreased accuracy at 84%. In order to overcome the constraints of barcode detection, we integrated an external scanner specifically designed for small vials. In the future, our objective is to integrate deep learning models to detect vials in real-time, with a specific emphasis on object detection approaches. Instead of relying on barcode data, these models will identify vials based on their visual attributes. Object identification algorithms can undergo training to identify different vial shapes and sizes, which has the potential to improve detection accuracy in difficult circumstances. Using deep learning for appearance-based detection could provide a more reliable method to identify vials, particularly in cases where barcodes are not easily observable. In addition, increased camera resolution and computer capabilities may further improve barcode detection.

## Figures and Tables

**Table 1.** Comparison of existing work and our MR-based detection system.

Method	Accuracy	Speed (Frames/s)	Detection Capability	Technologies Used	Challenges Addressed	Limitations
Method A (Traditional Barcode [10])	85%	10–20 fps	Simple Barcodes Only	Barcode Scanner	Works under standard conditions	Fail to scan 29% of medications and need manual handling of software.
Method B (Machine Learning [9,19])	89.3%	10–30 fps	Complex Patterns	CNN for Image Recognition	High accuracy on test datasets	Slower and struggles with real-time performance, High inference time.
**Our Method (MR + HoloLens 2)**	**98%**	**10.54–52.5 fps**	Real-time Barcode and Vial Detection	Mixed Reality, HoloLens 2 barcode, scanner	Real-time hands-free detection, better under dynamic conditions	Device cost, computational limitations, fail to detect below certain size by Hololens 2.

**Table 2.** Vial information and barcode size measurements (both real and fake medication vials were used).

Medicine Name	Barcode Size	Remarks
Lidocaine HCI	3.4 × 1 cm	Injection (paper box)
Calcium Chloride	3.4 × 1 cm	Injection (paper box)
Amiodron	1.2 × 0.5 cm	Single dose injection vial
Adenosin	1.2 × 0.4 cm	Injection 2 mL vial
Amiodarone HCI	1.6 × 0.4 cm	Injection 3 mL vial
Fentanl Citrat	0.9 × 0.4 cm	Single dose injection vial
Epinephrine	4.4 × 1.3 cm	Injection (paper box)
Atropine Sulfate	3.7 × 1.3 cm	Injection (paper box)

**Table 3.** Vial information and detection statistics.

Medicine Name	Barcode Size	Hololens 2 Camera	External Scanner	Average Distance (cm) and Time (ms) Avg. Barcode Processing Time + Avg. Information Retrieval Time
Lidocaine HCI	3.4 × 1 cm	Detected	Detected	22.86/57.6 + 305.5
Calcium Chloride	3.4 × 1 cm	Detected	Detected	22.5/57.7 + 315.5
Amiodron	1.2 × 0.5 cm	Not detected	Detected	–
Adenosin	1.2 × 0.4 cm	Not detected	Detected	–
Amiodarone HCI	1.6 × 0.4 cm	Not detected	Detected	–
Fentanyl Citrate	0.9 × 0.4 cm	Not detected	Detected	–
Epinephrine	4.4 × 1.3 cm	Detected	Detected	25.6/57.8 + 306.5
Atropine Sulfate	3.7 × 1.3 cm	Detected	Detected	23.78/60.3 + 310.6

**Table 4.** Frame rate measurements when camera resolution set for 1920 × 1080 for Hololens 2.

Test Condition	Average Frame Rate (fps)	Maximum Frame Rate (fps)	Minimum Frame Rate (fps)
Normal Lighting (room light-300 lux)	10.54	11.80	9.26
Low Lighting (dim light-50 lux)	10.10	11.22	9.13
High Barcode Density	10.05	11.33	9.57

**Table 5.** Resolution and accuracy trade-off using Hololens 2 camera.

Resolution	Detection Accuracy (%)	Average Processing Time (ms)	Frame Rate (fps)
1920 × 1080	98	60.3	10.54
1280 × 720	93	17.1	52.5
640 × 480	84	16.2	49.3

## Data Availability

The original contributions presented in the study are included in the article, further inquiries can be directed to the corresponding author.
